# Behind the scrubs: Psychological distress and resilience among nurses

**DOI:** 10.4102/hsag.v30i0.2820

**Published:** 2025-03-21

**Authors:** Jennifer Chipps, Ilze Steenkamp, Anita Padmanabhanunni, Petra Brysiewicz, Amanda Cromhout

**Affiliations:** 1 School of Nursing, Faculty of Community and Health Sciences, University of the Western Cape, Bellville, South Africa; 2 Department of Psychology, Faculty of Community and Health Sciences, University of the Western Cape, Bellville, South Africa; 3 School of Nursing & Public Health, College of Health Sciences, University of KwaZulu-Natal, Durban, South Africa

**Keywords:** categories of nurses, moral distress, sources of stress, fear of COVID-19, vulnerability to disease, psychological distress, resilience

## Abstract

**Background:**

Nurses are exposed to high levels of stress in the workplace. During the coronavirus disease 2019 (COVID-19) pandemic, levels of stress were exacerbated, impacting on nurses’ mental health.

**Aim:**

The aim of the study was to investigate psychological distress and resilience, and how nurses with different levels of education responded to stress.

**Setting:**

The study was conducted in three hospitals (a psychiatric hospital, a general district hospital and a dedicated COVID-19 hospital) in the Western Cape province, South Africa.

**Methods:**

A survey was conducted with frontline nurses (*N* = 167 [71.8%]) in three hospitals in the Western Cape using six validated self-administered scales.

**Results:**

Respondents reported high levels of moral distress related to time (3.42/6, ± 1.6) and protection during COVID-19 (1.3/3, ± 0.7). Mild-to-moderate levels of fear of COVID-19 (19.4/35, ± 8.2) and a moderate perception of vulnerability to disease (60.7/105, ± 19.9) contributed to nurses’ stress. High levels of psychological distress, especially during COVID-19 compared to current levels (27.2 vs 18.8; *W* = 8.9, *p* = < 0.001), with high levels of resilience (73.2/88, ± 17.9) were reported. Enrolled nurses reported significantly higher levels of stress during the pandemic.

**Conclusion:**

Post COVID-19, there was reduction in the respondents who reported severe levels of psychological distress, highlighting the impact of the pandemic on nurses’ mental health and the need to build resilience.

**Contribution:**

This study enhances understanding of the factors that result in psychological distress in nurses and how nurses with different levels of education respond to stress.

## Introduction

The shortage of nurses in South Africa has resulted in increased stress on nurses who already must cope with high workloads. Nursing is a high-stress profession, with increased vulnerability to disease (Gökkaya et al. 2022; Osagiator Ariyo et al. 2022; Padmanabhanunni & Pretorius 2023; Pasay-An 2020). This was especially experienced during coronavirus disease 2019 (COVID-19), with exposure to a contagious disease that was life-threatening (Van der Groot et al. 2021). In addition, nurses have to deal with daily ethical and moral dilemmas (Arafat, Tahir & Harisa 2023; Chersich et al. 2020; Lam & Hung 2013; Shanafelt, Ripp & Trockel 2020), as well as other work-related pressures (Wang et al. 2022), which can impact negatively on their mental health and well-being (Chipps & Jarvis 2021; Manzanares et al. 2021; Naidoo & Sibiya 2019).

The COVID-19 pandemic resulted in heightened stress, anxiety, burnout and moral distress in nurses (Brysiewicz & Chipps 2022; Manzanares et al. 2021; Riedel et al. 2022; Sun et al. 2020; Udwadia & Raju 2020), resulting in low job satisfaction, burnout and high turnover (Norman et al. 2021). Frontline workers across the globe reported symptoms of depression, anxiety and stress (Van der Groot et al. 2021), with post-traumatic stress being the most commonly reported mental health condition, followed by psychological distress (Al Maqbali, Al Sinani & Al-Lenjawi 2021; Saragih et al. 2021). Many nurses also considered leaving the profession (Maben et al. 2022). An additional concern was the impact of varying levels of preparation for the pandemic, expressed in the educational preparation of nurses in terms of different categories of nurses. A previous study with psychiatric nurses in South Africa found that enrolled nurses and nurse assistants reported lower levels of burnout and secondary traumatic stress than the other professional groups (Maila, Martin & Chipps 2020).

Responding to stress may result in negative and positive coping strategies. A study by Munyanziza et al. (2021) found that three quarters of Intensive Care Unit (ICU) nurses in Rwanda used alcohol or drugs to cope with stress during the pandemic. However, positive responses to stress, such as resilient active coping strategies, and social support were found to be associated with lower levels of psychological distress in the general Chinese population (Yu et al. 2020).

Resilience, as a response to stress, is a process where both assets (personal characteristics, e.g. coping skills) and resources (external protective factors, e.g. social support systems) are used to obtain positive outcomes (Fergus & Zimmerman 2005).

### Conceptual framework

The theory of a problem framework (Sidani & Branden 2021) is used to articulate the health problem that needs intervention. It describes the problem to identify factors that contribute to the problem – which can be predisposing, precipitating or perpetuating – and can specify possible consequences should the problem not be addressed. For this study, the identified problem was the high levels of stress that nurses are exposed to, for example COVID-19 and moral distress. Fear of COVID-19 was identified as a contributing precipitating factor, and perceived vulnerability of disease as the perpetuating factor. Psychological distress and resilience (measured as response to stress) were specified as possible outcomes of stress.

### Aim of the study

The aim of the study was to investigate factors contributing to and the consequences of a major stressor, namely COVID-19, in terms of psychological distress and resilience among nurses with different levels of educational preparation.

## Research methods and design

A quantitative descriptive cross-sectional study was conducted. A survey was used to collect the data from nurses in three hospitals in the Western Cape between October and December 2022, after the COVID-19 pandemic.

### Setting

The study was conducted in three hospitals (a psychiatric hospital, a general district hospital and a dedicated COVID-19 hospital) in the Western Cape province, South Africa. These hospitals were purposively selected to represent diverse hospital settings that dealt with a wide range of patients during COVID-19.

### Study population and sample

The study population were all categories of nurses working at the three hospitals. Using a pooled anxiety prevalence of 23.2% (Pappa et al. 2020) and a precision of 5%, a sample size of 271 was calculated. Inclusion criteria were nurses, registered and enrolled, with the South African Nursing Council (SANC), and involved in direct patient care. Purposive sampling was used to recruit the sample from the three hospitals described earlier.

### Instruments

A self-reported questionnaire was used to collect the data. The questionnaire included questions on gender, age, level of educational preparation (reflecting the different categories of nurses), years of experience as a nurse and area of current work. In addition, for the COVID-19 period, questions were asked about whether nurses worked with patients diagnosed with COVID-19 during the pandemic, whether they were tested and/or diagnosed with COVID-19 and their area of contact with COVID-19. Based on the theory of a problem framework (Sidani & Braden 2021), six validated self-administered scales were used to measure the variables of interest (Table 1). Respondents completed the Kessler Psychological Distress Scale (Kessler-10, Kessler et al. 2002) twice: firstly, on their experiences during COVID-19 and secondly, on their experiences based on the 30 days preceding the completion of the questionnaire.

**TABLE 1:** Scales used in the study.

Scale	Description of scale	Reliability
Moral Distress Scale (Eizenberg et al. 2009)	A validated 15-item scale used to measure moral distress in three areas, namely relationships, resources and time on a 6-point scale ranging from 1 (*not at all*) to 6 (*to a very large extent*), as well as a ‘not applicable’ option if the statement is not applicable. Scores can range between 15 and 90.	α = 0.84 (Eizenberg et al. 2009) α = 0.92^a^
Sources of Stress Scale (Shanafelt et al. 2020)	A validated 13-item scale that measures different sources of stress and/or anxiety (protect me, care for me, prepare me, hear me, support me) as experienced over the past month on a 3-point Likert scale.	α = 0.76 (Chipps et al. 2022) α = 0.90^a^
Fear of COVID Scale (FCV-19S) (Ahorsu et al. 2020)	A validated 7-item scale that measures the level of fear of COVID-19 on a 5-point Likert scale ranging from 1 (*strongly disagree*) to 5 (*strongly agree*). Total scores between 7 and 35 are possible, with higher scores indicating higher levels of fear of COVID-19.	α = 0.92 (Ahorsu et al. 2020) α = 0.90^a^
Perceived Vulnerability to Disease Scale (Duncan, Schaller and Park 2009)	A validated 15-item scale that measures one’s perception of one’s vulnerability to infectious disease on a 7-point scale ranging from 1 (*strongly disagree*) to 7 (*strongly agree*). The scale consists of two subscales, namely perceived infectability (7 items) and germ aversion (8 items). Possible scores between 7 and 105 are possible, with higher scores indicating higher levels of perceived vulnerability.	α = 0.71 (perceived infectability) α = 0.77 (germ aversion) (Padmanabhanunni et al. 2021) α = 0.73^a^ (perceived infectability) α = 0.90^a^ (germ aversion)
Kessler Psychological Distress Scale (Kessler-10; Kessler et al. 2002)	A validated 10-item scale used to measure psychological distress (scale 1 [*none of the time*] to 5 [*all of the time*]) in the last 30 days and during the pandemic. Scores range between 10 and 50, with higher scores indicating higher levels of psychological distress and the possibility of mental health problems.	α = 0.86 (Slade, Grove & Burgess 2011) α = 0.95 during pandemic^a^ α = 0.93 after pandemic^a^
Response to Stressful Event Scale (Johnson et al. 2011)	A validated 22-item scale that measures different responses to stressful events on a 5-point scale ranging from 0 (*not at all like me*) to 4 (*exactly like me*). The scale has five subscales, namely active coping, meaning-making, spirituality, self-efficacy and cognitive flexibility. Total scores range between 0 and 88, with higher scores indicating higher levels of resilience.	α = 0.86 (Steenkamp & Chipps 2024) α = 0.90^a^

Note: Please refer to the reference list for full details of the references cited in the table.

COVID-19, coronavirus disease 2019.

a , Cronbach’s alpha for this study.

### Data collection

The nurse managers of each of the selected hospitals were contacted via email, and arrangements were made to facilitate access to potential respondents. Four trained clinical facilitators collected the data between October 2022 and December 2022, with ongoing cases of COVID-19 in healthcare facilities in South Africa. The respondents were informed of the purpose of the study and that participation was voluntary. Written informed consent was obtained from those who agreed to participate. Questionnaires were paper-based and took approximately 15 min to complete.

### Data analysis

Statistical analysis was performed using the IBM SPSS v 28.0 (IBM Corp. Released 2021. IBM SPSS Statistics for Windows, Version 28.0. Armonk, NY: IBM Corp). Frequencies and descriptive statistics were used to describe the demographic data, and differences between nurse categories and demographic data were determined using Chi-square tests. The results were analysed according to nurses’ level of educational preparation (category of nurse). Non-parametric tests were used to compare the contributing factors to psychological distress among the different categories of nurses. Logistic regression was performed to assess the impact of several factors on psychological distress during COVID-19 and the last 30 days, prior to completing the questionnaire. The scales were analysed according to the instructions for each scale:

- *Moral distress:* Scale statements were averaged for each of the three domains, namely Relationships, Resources and Time, and summed to derive a total score. If the score reached or exceeded 50% of the maximum possible score, it was regarded as perpetuating psychological distress (Eizenberg et al. 2009). Moral distress was recoded into a binary variable reflecting not having moral distress (low levels of moral distress: scores between 15 and 30) and having moral distress (including moderate and high levels of distress with scores between 31 and 60 [moderate] and 61 and 90 [severe]).
- *Source of stress*: Individual mean scores of the scale statements and the domains of the scale (Shanafelt et al. 2020) were calculated and ranked from highest source of anxiety to the lowest (Chipps et al. 2022).
- *Fear of COVID-19*: Ratings on the Fear of COVID Scale (FCV-19 scale) statements were summed to derive a total score (Ahorsu et al. 2020). Scores of 7 and above indicated the presence of fear of COVID-19, with categories of severity indicated as mild (scores between 7 and 19), moderate (scores between 20 and 26) and severe (scores of 27 and above) (Faro et al. 2022).
- *Vulnerability to disease*: The scale statements were summed to derive a score for the two subscales, perceived infectability and germ aversion. If the score reached or exceeded 50% of the maximum possible score for each subscale, it was regarded as perpetuating psychological distress (Duncan et al. 2009).
- *Kessler-10*: Ratings on the Kessler-10 were summed to derive a total score. Respondents were classified as ‘likely to be well’ (scores below 20) or likely to have mild (scores between 20 and 24), moderate (scores between 25 and 29) or severe (scores of 30 and above) psychological distress (Kessler et al. 2002). Psychological distress scores during COVID-19 and the last 30 days, prior to completing the questionnaire, were compared using Wilcoxon Signed Rank Test. Psychological distress was recoded into binary variable, reflecting no distress (scores below 20) and having distress (including mild [scores between 20 and 24], moderate [scores between 25 and 29], or high [scores of 30 or above] levels of distress).
- *Response to stressful events*: Ratings on the Response to Stressful Event Scale were summed to derive a total score. Respondents were regarded as having low (scores below 49), moderate (scores between 50 and 70) and high (scores between 71 and 88) resilience based on their total score (Johnson et al. 2011). Means were calculated for the subscales.

### Ethical considerations

The study was approved by the Institutional Review Board at a large public university in the Western Cape (reference number: BM20/6/2) and the South African National Health Research Database (WC_202012_021). Permission to conduct the study was obtained from the Chief Executive Officers of the selected hospitals. Information about the study was distributed to all respondents. Written informed consent was obtained from those who agreed to participate. Participation was voluntary and questionnaires were completed anonymously, and confidentiality was maintained.

## Results

### Demographics and sample realisation

A total of 231 questionnaires were distributed to nurses across the three hospitals and 167 questionnaires were completed (71.8% response rate). The average age of the respondents was 39.8 (± 10.4) years. Most of the respondents were female (146, 88%). On average, respondents had 11.9 (± 10.8) years of work experience (median = 8, range: 1–40 years). More than half of the respondents were professional registered nurses (*n* = 87, 52.1%), followed by enrolled nursing auxiliaries (*n* = 59, 35.2%) and enrolled nurses (*n* = 19, 11.4%). There were significant differences between the categories of nurses in terms of gender (no male enrolled nurses) (*χ*^2^ = 17.4, *p* < 0.001; Table 2). Almost half of the respondents worked in psychiatric units (*n* = 81, 49.7%), followed by general areas (medical, surgical or orthopaedic) (*n* = 32, 19.6%), and eight (4.9%) respondents worked in emergency departments. There were significant differences in work area by category of nurse, with general areas being the most frequent work area for enrolled nurses and enrolled nursing auxiliaries (*χ*^2^ = 32.6, *p* = <0.001; Table 2).

**TABLE 2:** Demographic variables and COVID-19 exposure of nurses by total sample and category of nursing.

Variables	Total sample (*n* = 167)				Professional nurses (*n* = 87)				Enrolled nurses (*n* = 19)				Enrolled nursing auxiliaries (*n* = 59)				Test	*p*
	*n*	%	Mean	s.d.	*n*	%	Mean	s.d.	*n*	%	Mean	s.d.	*n*	%	Mean	s.d.		
**Gender**	-	-	-	-	-	-	-	-	-	-	-	-	-	-	-	-	^*χ*2^ = 17.4	< 0.001*
Male	20	12.0	-	-	15	17.2	-	-	5	26.3	-	-	0	0	-	-	-	-
Female	146	88.0	-	-	71	81.6	-	-	14	73.7	-	-	59	100	-	-	-	-
Age	-	-	39.8	±10.42	-	-	38.3	±11.2	-	-	42.9	±9.6	-	-	40.8	±9.1	*K* = 4.5	0.106
Experience (years)	-	-	11.9	±10.82	-	-	11.9	±11.3	-	-	14.5	±12.6	-	-	10.7	±9.0	*K* = 1.4	0.495
**Area of current work**	-	-	-	-	-	-	-	-	-	-	-	-	-	-	-	-	^*χ*2^ = 32.6	< 0.001*
Midwifery	9	5.5	-	-	8	9.3	-	-	0	0	-	-	1	1.8	-	-	-	-
Paediatrics	12	7.4	-	-	3	3.5	-	-	2	11.1	-	-	6	10.5	-	-	-	-
Critical care	9	5.5	-	-	2	2.3	-	-	0	0	-	-	7	12.3	-	-	-	-
General	32	19.6	-	-	11	12.8	-	-	5	27.8	-	-	16	28.1	-	-	-	-
Psychiatric	81	49.7	-	-	56	65.1	-	-	7	38.9	-	-	17	29.8	-	-	-	-
Emergency	8	4.9	-	-	2	2.3	-	-	2	11.1	-	-	4	7.0	-	-	-	-
Other	12	7.4	-	-	4	4.7	-	-	2	11.1	-	-	6	10.5	-	-	-	-
Worked with COVID-19 patients	160	97.6	-	-	85	98.8	-	-	17	89.5	-	-	56	98.2	-	-	^*χ*2^ = 4.4	0.102
**COVID-19**																		
Tested	82	49.1	-	-	51	58.6	-	-	11	57.9	-	-	19	32.2	-	-	^*χ*2^ = 10.5	0.006*
Diagnosed	57	34.1	-	-	33	37.9	-	-	5	26.3	-	-	18	30.5	-	-	^*χ*2^ = 1.4	0.488
**Area of contact**																		
Screening of patients	38	22.8	-	-	21	24.1	-	-	7	36.8	-	-	10	16.9	-	-	^*χ*2^ = 3.3	0.198
General unit	140	83.8	-	-	74	85.1	-	-	12	66.3	-	-	52	88.1	-	-	^*χ*2^ = 6.9	0.031
Critical care unit	29	17.4	-	-	11	12.6	-	-	5	26.5	-	-	13	22.0	-	-	^*χ*2^ = 3.3	0.198
Emergency unit	20	12.0	-	-	8	9.2	-	-	1	5.3	-	-	11	18.6	-	-	^*χ*2^ = 3.9	0.161
Other	4	2.4	-	-	4	4.6	-	-	0	0	-	-	0	0	-	-	^*χ*2^ = 2.6	0.244

Note: Missing values were not included.

s.d., standard deviation; *χ*^2^, Chi-square test or Fisher’s exact test where relevant; *K*, independent Kruskal–Wallis test; COVID-19, coronavirus disease 2019.

* , *p* < 0.05.

### Exposure to COVID-19

Nearly all the respondents worked with COVID-19 patients during the pandemic (*n* = 160/167 [97.6%]). Almost half of the respondents (*n* = 82, 49.1%) were tested for COVID-19, and over a third (*n* = 57, 34.1%) were diagnosed with COVID-19. More professional nurses were tested for COVID-19 than enrolled nurses and enrolled nursing auxiliaries (58.6% vs. 57.9% and 32.2%, respectively, *χ*^2^ = 10.5, *p* = 0.006). Most contact with COVID-19 patients were in general units (*n* = 140, 83.8%), followed by the screening of patients (*n* = 38, 22.8%). Only 12 (7.4%) respondents reported having contact with COVID-19 in paediatric units or other places (*n* = 12, 7.4%; Table 2).

### Moral distress and sources of stress during COVID-19

*Moral distress*: A total of 149 respondents completed the scale. The average score for moral distress among respondents was 24/90 (± 18.78). The average score for the total sample and across the different categories of nurses was less than 50% of the total maximum score and therefore did not contribute to psychological distress. There were no statistically significant differences in the moral distress score among the different categories of nurses (*K* = 0.036, *p* = 0.982). The lack of time (2.27/6 ± 1.6) was rated significantly higher than the lack of resources (1.57/6 ± 1.4) or relationship challenges (1.55/6 ± 1.5) with no significant differences between the categories of nurses (Table 3).

**TABLE 3:** Scales per nurse categories.

Variables	Total sample (*n* = 167)				Professional nurses (*n* = 87)				Enrolled nurses (*n* = 19)				Enrolled nursing auxiliary (*n* = 59)				Test	*n*
	Mean	s.d.	*n*	%	Mean	s.d.	*n*	%	Mean	s.d.	*n*	%	Mean	s.d.	*n*	%		
**The problem**																		
*Moral distress/66*	24.0	±18.78	-	-	24.32	±18.44	-	-	22.81	±14.0	-	-	24.90	±21.14	-	-	*K* = 0.04	0.982
Lack of time/6	2.27	±1.16	-	-	2.15	±1.71	-	-	2.23	±0.82	-	-	2.51	±1.60	-	-	*K* = 2.5	0.292
Relationships/6	1.60	±1.43	-	-	1.52	±1.29	-	-	1.58	±1.32	-	-	1.79	±1.66	-	-	*K* = 0.2	0.907
Lack of resources/6	1.51	±1.27	-	-	1.44	±1.17	-	-	1.35	±0.93	-	-	1.7	±1.50	-	-	*K* = 0.3	0.850
*Sources of stress/3*																		
Protection	1.3	±0.7	-	-	1.4	±0.6	-	-	1.6	±0.8	-	-	1.2	±0.7	-	-	*K* = 7.2	0.027*
Care	1.3	±0.9	-	-	1.3	±0.9	-	-	1.6	±1.1	-	-	1.1	±0.8	-	-	*K* = 3.0	0.222
Preparation	1.2	±0.9	-	-	1.1	±0.8	-	-	1.5	±0.9	-	-	1.3	±0.9	-	-	*K* = 4.1	0.130
Consideration	1.0	±1.0	-	-	0.9	±1.0	-	-	1.5	±1.1	-	-	1.2	±1.0	-	-	*K* = 7.1	0.029*
Support	1.0	±0.9	-	-	1.0	±0.9	-	-	1.4	±0.9	-	-	1.0	±0.9	-	-	*K* = 5.2	0.074
**Contributing (precipitating) factors**																		
*COVID-19 fear/35*	19.4	±8.4	-	-	17.9	±7.6	-	-	20.26	±10.6	-	-	21.35	±8.5	-	-	*K* = 5.1	0.080
Mild	-	-	58	34.7	-	-	35	42.7	-	-	8	42.1	-	-	15	27.8	^*χ*2^ = 10.2	0.037*
Moderate	-	-	56	33.5	-	-	32	39.0	-	-	3	15.8	-	-	19	35.2		
Severe	-	-	43	25.7	-	-	15	18.3	-	-	8	42.1	-	-	20	37.0		
*Perceived vulnerability to disease*/105	60.7	±19.9	-	-	58.1	±18.9	-	-	67.2	±24.3	-	-	62.4	±19.5	-	-	*K* = 1.98	0.372
Perceived infectability/49	27.0	±10.5	-	-	25.8	±9.5	-	-	28.3	±12.8	-	-	28.2	±10.9	-	-	*K* = 0.60	0.739
Germ aversion/56	32.1	±11.2	-	-	31.9	±10.7	-	-	36.6	±13.3	-	-	31.8	±11.3	-	-	*K* = 1.2	0.542
**Outcomes**																		
Psychological distress score during COVID-19/50	27.2	±11.6	-	-	24.2	±9.6	-	-	31.2	±11.3	-	-	30.9	±13.1	-	-	*K* = 10.9	0.004*
Psychological distress score in the last 30 days/50	18.8	±8.9	-	-	17.6	±7.6	-	-	22.8	±9.9	-	-	19.5	±10.1	-	-	*K* = 3.9	0.144
Response to stress score/88	73.2	±17.9	-	-	74.9	±13.2	-	-	68.0	±26.8	-	-	72.7	±20.2	-	-	*K* = 0.5	0.800

Note: Missing values were not included.

COVID-19, coronavirus disease 2019; s.d., standard deviation; *χ*^2^, Chi-square test or Fisher’s exact test where relevant; *K*, independent Kruskal–Wallis test.

* , *p* < 0.05.

*Sources of stress:* Sources of stress were classified into five domains in terms of stress related to protection, preparation, consideration, support and care. Stress related to *protection* (1.3, ± 0.7) had the highest mean score, followed by *care* (1.3, ± 0.9), *preparation* (1.2, ± 0.9), *consideration* (1.0, ± 1.0) and *support* (1.0, ± 0.9) (Table 3). Professional nurses had higher stress scores for *protection* (1.6 vs. 1.4 vs. 1.2, *K* = 7.2, *p* = 0.027) and *consideration* (1.5 vs. 1.2 vs. 0.9, *K* = 7.1, *p* = 0.029) compared to enrolled nurses and enrolled nurse assistants (Table 3). Specific stress related to *exposure at work* (1.6/3, ± 0.9) and *stress about infecting family at home* (1.5/3, ± 0.9) were rated the highest. *The lack of access to up-to-date information* (1.8 vs. 0.9 vs. 1.1, *K* = 6.3, *p* = 0.043) and *the lack of access to up-to-date communication* were scored higher among enrolled nurses compared to professional nurses and enrolled nursing auxiliaries (1.7 vs. 0.9 vs. 1.2, *K* = 7.3, *p* = 0.026; Table 3).

### Fear of COVID-19

Respondents reported mild-to-moderate fear of COVID-19 (19.4/35, ± 8.4). Fewer professional nurses were classified as having severe fear of COVID-19 (15, 18.3%) compared to enrolled nurses (8, 42.1%) and enrolled nursing auxiliaries (20, 37%) (*χ*^2^ = 10.2, *p* = 0.037; Table 3).

### Vulnerability to disease

Respondents reported a mean score of 60.7/105 (± 19.9) for the total scale, with higher scores indicating greater perceived vulnerability. Enrolled nurses reported the highest level of perceived vulnerability (67.2, ± 24.3) and professional nurses the lowest level (58.1, ± 18.9; *K* = 1.98, *p* = 0.372). For *perceived infectability*, respondents reported a mean score of 27/49 (± 10.5) and for *germ aversion,* a mean score of 32.1/56 (± 11.2), with higher scores reflecting higher discomfort with potential disease transmission (Padmanabhanunni & Pretorius 2023; Pretorius et al. 2022). Total and subscale scores exceeded the maximum possible score for nurses of all levels of educational preparation and therefore contribute to psychological distress. There were no significant differences between the different categories of nurses (Table 3).

### Psychological distress

**FIGURE 1:**
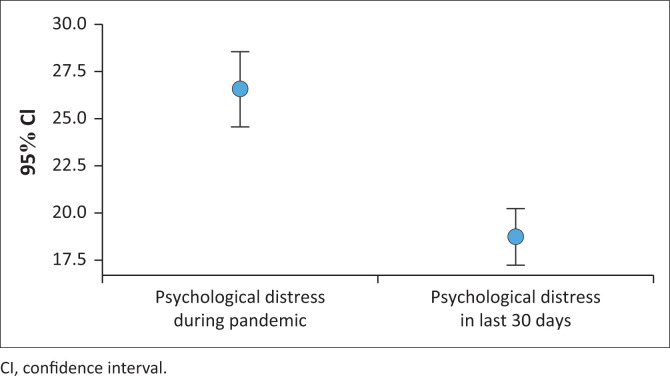
Psychological distress during COVID-19 and last 30 days.

Psychological distress was significantly higher during COVID-19 (27.2, ± 11.6) compared to the last 30 days prior to completing the questionnaire (18.8, ± 8.6; *W* = 8.9, *p* = <0.001; Table 3). This was true for nurses with all levels of educational preparation. However, in the last 30 days prior to completing the questionnaire, enrolled nurses reported the highest levels of psychological distress (during the pandemic: 31.2, ± 11.3 and last 30 days: 22.8, ± 9.9, respectively) compared to professional nurses who reported the lowest level of psychological distress during the pandemic (24.2, ± 9.6) and in last 30 days (17.6, ± 7.6; *K* = 10.9, *p* = 0.004) (Table 3, Figure 1). Psychological distress was moderately correlated with fear of COVID-19 (*r* = 0.478, *p* < 0.001) and weakly with moral distress (*r* = 0.28, *p* = 0.003) and perceived vulnerability to disease (*r* = 0.201, *p* = 0.04) during the last 30 days prior to completing the questionnaire; and during the pandemic highly with fear of COVID-19 (*r* = 0.502, *p* < 0.001) and moderately with moral distress (*r* = 0.350, *p* < 0.001).

Direct logistic regression was performed to assess the impact of several factors on the likelihood that respondents meet criteria for psychological distress (moderate or severe stress) in the last 30 days prior to completing the questionnaire and during COVID-19.

The model of last 30 days prior to completing the questionnaire contained three independent variables (level of educational training, perceived vulnerability to disease, moral distress). The full model containing all predictors was statistically significant, *X*^2^ (3, *N* = 167) = 16.7, *p* < 0.002, indicating that the model was able to distinguish between respondents who were distressed and not distressed. The model explained between 19.7% (Cox and Snell R square) and 26.3% (Nagelkerke R squared) of the variance and correctly classified 69.7% of cases. One independent variable made a unique statistically significant contribution to the model, with moral distress making the biggest contribution, recording an odds ratio of 2.4–21.4 (*p* < 0.001). Nearing significance (as a result of small sample size) was being an enrolled nurse (OR: 0.93–22.1, *p* = 0.062).

The distress during COVID-19 model contained four independent variables (level of educational training, perceived vulnerability to disease, moral distress and COVID-19 fear). The full model containing all predictors was statistically significant, *X*^2^ (4, *N* = 167) = 15.8 *p* < 0.001, indicating that the model was able to distinguish between respondents who were distressed and not distressed. The model explained between 18.1% (Cox and Snell R square) and 26.5% (Nagelkerke R squared) of the variance and correctly classified 77.9% of cases. One independent variable made a unique statistically significant contribution to the model, namely, moral distress – lack of time (OR: 1.6–25.9, *p* = 0.008). Having severe fear of COVID-19 (OR: 0.78–29.7, *p* = 0.091) approached significance.

#### Response to stress (resilience)

Overall, the respondents reported high levels of resilience (73.2/88, ± 17.9), with 98 (58.7%) classified as having high levels of resilience, 34 (20.4%) as moderate level of resilience and 12 (7.2%) as low level of resilience. Professional nurses (74.9, ± 13.2) and enrolled nursing auxiliaries (72.7, ± 20.2) reported high levels of resilience, and enrolled nurses reported moderate levels of resilience (68.0, ± 72.7, *K* = 0.45, *p* = 0.800; Table 3). The highest rated coping strategy was *spirituality* (3.44, ± 0.97), followed by *active coping* (3.31, ± 0.79), *meaning-making* (3.29, ± 0.99), *self-efficacy* (3.24, ± 0.99) and *cognitive flexibility* (3.15, ± 1.0), with no significant difference.

## Discussion

The aim of the study was to investigate factors contributing to and the consequences of a major stressor, such as COVID-19, in terms of psychological distress and resilience among nurses with different levels of educational preparation. Overall, the study found low levels of distress in the nurse respondents (18.8/50) in the last 30 days prior to completing the questionnaire, with the score falling in the *likely to be well* category (Spies et al. 2009). However, an extreme stressor, such as COVID-19, had a significant impact on stress levels, with all respondents reporting significantly higher levels of psychological distress during the pandemic (27.2/50). The number of nurses who were likely to be well more than doubled after the pandemic from 45 (26.9%) in the pandemic to 89 (53.3%) post-pandemic (McNemar–Bowker test = 66.5, *p* ≤ 0.001), with a notable reduction in the number of respondents who were likely to suffer from a severe mental disorder. This result is expected, as the normal stressors that nurses encounter daily were exacerbated during the COVID-19 pandemic, when work and other demands were higher than usual, with nurses having to work extra shifts, function in new roles, and adapt to new situations and protocols (Van der Groot et al. 2021). The result is in line with previous research where frontline workers across the globe reported symptoms of depression, anxiety and stress during the pandemic (Van der Groot et al. 2021).

Several factors were found to contribute to nurses’ levels of distress. In the last 30 days prior to completing the questionnaire, moral distress, primarily because of a lack of time, perceived vulnerability to disease and different levels of educational preparation (specifically being an enrolled nurse with 2 years of training) all contributed to moderate or severe distress levels. During COVID-19, moral distress and fear of COVID-19 significantly contributed to moderate or severe distress levels. Moral distress, the psychological experience in response to moral stressors, such as a lack of time because of staff shortages, is common in the nursing profession (Eşer et al. 2021; Riedel et al. 2022). Studies have shown that moral distress increased during the pandemic, with increased reports of post-traumatic stress, burnout and high turnover of staff (Arafat et al. 2023; Norman et al. 2021; Silverman et al. 2021).

This is compounded with beliefs of perceived vulnerability, with our study finding moderate scores (60.7/105, ± 19.9). Coronavirus disease 2019 is a life-threatening disease, and although nurses in this sample reported mild-to-moderate (19.4/35, ± 8.2) levels of fear of contracting COVID-19, they may have felt a heightened sense of vulnerability to contract the disease considering that the disease is highly contagious. Several studies found that the COVID-19 pandemic impacted on nurses’ perceived vulnerability to the disease (Gökkaya et al. 2022; Osagiator Ariyo et al. 2022; Padmanabhanunni & Pretorius 2023; Pasay-An 2020) and could result in heightened stress, anxiety, burnout and moral distress (Brysiewicz & Chipps 2022; Manzanares et al. 2021; Riedel et al. 2022; Sun et al. 2020; Udwadia & Raju 2020). Nurses’ sense of vulnerability may further have been exacerbated by realities of workload and time constraints, limited access to personal protective equipment (PPE), access to timely information, and fears of infecting others or family members at home. In the current sample, nurses reported higher levels of stress related to stressors of *protection* (1.3, ± 0.7) and *care* (1.3, ± 0.9), with professional nurses having higher stress scores for *protection* (1.6 vs. 1.4 vs. 1.2, *K* = 7.2, *p* = 0.027) and *consideration* (1.5 vs. 1.2 vs. 0.9, *K* = 7.1, *p* = 0.029) compared to enrolled nurses and enrolled nursing auxiliaries.

The finding also confirmed that the educational preparation of nurses in terms of different categories of nurses could negatively impact their sense of self-efficacy in dealing with stress, with enrolled nurses reporting significantly higher stress and vulnerability. Possibly because of increased stress during the pandemic, these findings are in contrast to a previous study in South Africa in a psychiatric setting, which found enrolled nurses and nursing auxiliaries with lower levels of burnout and secondary traumatic stress than the other professional groups (Maila et al. 2020).

The nursing profession is exposed to several stressors related to the profession, and an event like COVID-19 can significantly impact on nurses’ mental health, although the respondents reported moderate levels of resilience. This was similar to another South African study of nursing students during COVID-19 (Steenkamp & Chipps 2024) and as reported globally (Brysiewicz & Chipps 2022). Respondents in this study used a variety of (positive) coping strategies, including active coping, meaning-making, self-efficacy and cognitive flexibility and relying on spirituality. This was again similar to the South African nursing student study, which found spirituality rated the highest (3.36/4, ± 0.88), followed by self-efficacy (3.34/4, ± 0.64), meaning-making (3.26/4, ± 0.56), active coping (3.24/4, ± 0.66) and cognitive flexibility (3.05/4, ± 0.92), with no significant differences between these strategies (Steenkamp & Chipps 2024). Active coping strategies and increased social support are associated with lower levels of psychological distress and psychological capital (self-efficacy, hope, resilience, optimism) during COVID-19 (Sun et al. 2020). Nurses affected by stress require education, support and coping tools to alleviate the adverse effects on their well-being (Hossain & Clatty 2021). In addition, considering the role of social support in resilience and coping, the principles of Ubuntu that emphasise support, shared humanity and interconnectedness (Nicolaides 2023) can be useful to promote the social, physical, spiritual and psychological well-being of healthcare workers (Mulaudzi, Mogale & Masoga 2018; Nicolaides 2023; Rasweswe et al. 2024).

### Limitations of the study

The sample was drawn from three public hospitals in the Western Cape province and may not reflect the entire nurse population. Results may have varied for nurses working in private hospitals. In addition, data were collected post COVID-19, because of inability to access nurses during COVID-19, and recall bias may affect the findings. Lastly, it would have been beneficial to also measure the levels of moral distress during and after the pandemic.

### Recommendations

The nursing profession is, by nature, a high-stress environment, which can impact negatively on nurses’ mental health. Exposing nurses to resilience training programmes and teaching effective coping strategies may be useful in helping nurses cope with the burden of care. In addition, resilience training should include building self-efficacy, that is, adequately preparing nurses to deal with stressors in the workplace.

## Conclusion

Exposure to extreme stressors, such as the COVID-19 pandemic, and the concomitant impact on moral distress, especially related to time, can place additional stress on nurses and can impact on nurses’ mental health. However, despite this, nurses reported high levels of resilience, using a variety of coping strategies. Recognising this, there is a need to build on nurses’ natural resilience and positive responses to stress, with a specific focus on the educational preparation of the nurses in relation to the changed roles during crises.
